# Importance of Sensitive Vascular Measurements for Evaluating Effects of Lifestyle in Premenopausal Women

**DOI:** 10.3389/fcvm.2020.575908

**Published:** 2020-09-29

**Authors:** Abbi D. Lane-Cordova, Samantha Bouknight

**Affiliations:** ^1^Department of Exercise Science, Arnold School of Public Health, University of South Carolina, Columbia, SC, United States; ^2^Cardiovascular Translational Research Center, University of South Carolina School of Medicine, University of South Carolina, Columbia, SC, United States; ^3^Department of Pharmacology, Physiology, and Neuroscience, School of Medicine, University of South Carolina, Columbia, SC, United States

**Keywords:** hormones, premenopausal women, arterial stiffness, subclinical atherosclerosis parameter, lifestyle and behavior

## Abstract

Premenopausal women generally have a favorable cardiovascular risk profile, owing to young age and the protective effects of estrogen. Rates of hypertension and more advanced cardiovascular disease (CVD) are low in premenopausal women. A large body of epidemiological evidence has shown that lifestyle behaviors in midlife, i.e., cardiorespiratory fitness, physical activity, and healthy diet, are associated with lower risk of overt CVD and adverse cardiovascular outcomes in the future for men and women. Despite differences in future cardiovascular risk, brachial blood pressures might be similar between premenopausal women with favorable vs. unfavorable levels of lifestyle behaviors in early-to-mid-life. Here we make the case for deeper phenotyping by means of vascular function measurements, such as arterial stiffness, augmentation index, and endothelial function, to identify potential mechanistic pathways linking lifestyle behaviors in early-to-mid-adulthood with lifelong CVD risk in women. We describe considerations for vascular function measurement in premenopausal women and opportunities for investigators to fill in knowledge gaps to further our understanding of CVD risk assessment and CVD progression in premenopausal women.

## Introduction

Lifestyle interventions sometimes do not elicit significant improvements in brachial blood pressure (BP) in premenopausal women (1, 2). Reasons for the lack of improvement in BP or other traditional cardiovascular disease (CVD) risk factors after behavioral interventions are typically attributed to the general good cardiovascular health of premenopausal women due to younger age and protective effects of ovarian hormones (3). Despite the occasionally null or small effect on some traditional CVD risk factors, i.e., BP, lipids, and body mass index (BMI), following some interventions, lifestyle behaviors in young and middle adulthood have been consistently associated with CVD and traditional CVD risk factors later in life (4–6). In fact, improving any lifestyle behavior or CVD risk factor during adulthood has been linked to lower likelihood of subclinical atherosclerosis in middle age, underscoring the importance of lifestyle for longer-term CVD risk (7).

We propose that more sensitive measures of vascular structure and function are needed to understand how lifestyle influences CVD risk and better evaluate the effects of lifestyle behaviors and lifestyle interventions in premenopausal women. Some studies that employed more sensitive vascular measurements pre- and post- exercise intervention, such as central BP, arterial stiffness and distensibility, carotid intima-media thickness, or ventricular-vascular coupling, reported improvements in some of these measures while brachial BP stayed the same (1, 2, 8–11). Interestingly, changes in different vascular measurements were detected after different types of exercise training (indoor cycling, endurance, or circuit style resistance training), suggesting that subclinical vascular adaptations to exercise might be modality specific. These data illustrate the need for more thorough vascular characterization in larger samples of premenopausal women after different types of exercise interventions (1, 2, 8–11). Effects of endogenous and exogenous ovarian hormones should also be evaluated or accounted for, as some hormones have been linked to vascular structure and function and CVD risk (Table 1) and can be measured in clinic and research settings.

**Table 1:** Effects of ovarian hormones on vascular function measurements.

**Ovarian hormone**	**Association with vascular measurements**	**Reference**
Estrogen	↓brachial and central BP, ↑vascular reactivity	(12–16)
Progesterone	↓augmentation index, ↓vasoconstriction in response to agonists	(3, 17)
OCP	↑brachial and central BP during non-placebo phases vs. women not using OCP	(15, 18, 19)
AMH	↓in POI, ↑in PCOS, ↓carotid intima-media thickness in POI	(20–23)

*BP, blood pressure; OCP, oral contraceptive pill; AMH, anti-Müllerian hormone, a biomarker of ovarian reserve; POI, primary ovarian insufficiency; PCOS, polycystic ovarian syndrome. Female hormones have been linked to vascular structure and function and CVD risk and can be easily measured in clinic. Higher levels of estrogen and progesterone tend to have beneficial effects on the vasculature. Associations of AMH with vascular structure are contextual and may vary between women with POI or PCOS vs. controls*.

## Rationale for Collection of Sensitive Measurements of Vascular Structure and Function in Lifestyle Interventions

Ascertainment of hard outcomes, i.e., cardiovascular events, morbidity, and mortality, can guide clinical decision making and are used for evaluation of intervention success (24). However, trials designed with such hard outcomes as endpoints require significant time and resources. Other major clinical trials have used traditional CVD risk factors as endpoints (25, 26). Utilizing more proximal outcomes that are on the pathway toward hard outcomes and traditional CVD risk factors, such as less favorable vascular structure and function, as endpoints is attractive (24, 27). Trials designed with these proximal outcomes as endpoints may reduce the numbers of participants required, length of follow-up, and costs of the trial (24). Furthermore, more proximal outcomes, like some measures of vascular structure, and function, might be more malleable to change in response to lifestyle interventions than traditional CVD risk factors and used to gauge the success or potential for such interventions to interrupt the progression toward CVD risk factors or overt CVD (24, 27). The tracking of vascular functional measurements might be particularly important for younger women because there may be abnormalities in these measurements even when CVD risk factors are at normal levels.

With the exception of coronary artery calcium, the value of novel or subclinical CVD risk factors, such as poor endothelial function (measured using flow-mediated dilation), systemic inflammation (c-reactive protein), and ankle-brachial index, beyond traditional CVD risk factors for identifying individuals at high-risk for atherosclerotic CVD endpoints has not been consistently supported (28, 29). However, unlike the Framingham Risk Score used in earlier studies, more recently developed risk prediction algorithms for atherosclerotic CVD include race and/or inflammation to estimate CVD risk in women (30, 31). Guidance from the Pooled Cohort working group suggested that, although the utility of c-reactive protein for assessing CVD risk requires further study and was not included in the primary algorithm, clinicians may consider c-reactive protein levels to guide preventive strategies if a risk-score based treatment decision is unclear (30).

Importantly, the atherosclerotic CVD risk prediction models were calibrated to identify patients at risk for atherosclerotic, but not myocardial, CVD (30). A new risk prediction model calibrated to detect heart failure with preserved ejection fraction (HEFpEF; a myocardial disease), which occurs more frequently in women, is distinct from the atherosclerotic models in that it includes BMI and QRS interval in its algorithm (32). High BMI is a strong risk factor for HEFpEF in women (5). Further, BMI is consistently linked to vascular structure and function throughout the lifespan (33, 34), and the increased rate of aortic stiffening seen in obese adults in a community-based sample was partially attributed to systemic inflammation (33). Thus, the use of sensitive vascular-related measurements that have been related to vascular structure and physiological function, such as c-reactive protein, endothelial function, or arterial stiffness, as trial endpoints that might help reclassify women at intermediate risk for HEFpEF holds promise.

## Considerations for Data Collection in Premenopausal Women

There are challenges associated with collection of vascular function data in premenopausal women. Premenopausal women experience cycling of reproductive hormones, which affect some vascular function measurements, summarized in Table 1 (35). Estrogen and progesterone have dilating and anti-constricting vascular effects, respectively (3). One study reported lower brachial and central BP and higher vascular reactivity, measured using brachial artery flow-mediated dilation and calf and forearm reactive hyperemia, during the late follicular phase of menstrual cycle, i.e., days 7–14, that immediately precede ovulation and are characterized by a rapid rise in estrogen (12). Augmented brachial flow-mediated dilation and endothelium-independent dilation, assessed by determining dilation in response to sodium nitroprusside, were also observed during the late follicular phase of menstrual cycle in a prior study (13). In contrast, three more recent studies found there was no change in brachial flow-mediated dilation across phases of the menstrual cycle at rest (14, 15) or during handgrip exercise (16). More studies are needed in larger samples and diverse women to clarify the issue; earlier studies either did not report race or reported mostly Caucasian participants with mean BMI under 30 kg/m^2^. Augmentation index has been shown to be lower in the late luteal phase when progesterone is at its highest and estrogen is above the levels found in the early follicular phase (17). Beta-stiffness of the carotid artery and central pulse wave velocity were similar throughout the natural menstrual cycle in a recent study that included 33 women (18).

Use of oral contraceptive pills (OCP) might further complicate vascular assessment in premenopausal women and requires thoughtful consideration. While arterial stiffness was similar in women who do and do not use OCP, brachial and central BPs were higher during the non-placebo phases of the pill cycle in women who use OCP (15, 18, 19). Accordingly, the current standard is to account for phase of menstrual cycle or conduct all vascular assessment in the early follicular phase, i.e., days 1–7, when all reproductive hormones are at their lowest points (35, 36). This practice aims to standardize hormone levels, even in women who use cyclic OCP. However, there are multiple hormone preparations on the market, and little is known about how type and duration of OCP use influences measures of vascular function. Thus, further studies and thoughtfully collected data related to these topics are needed.

Premenopausal women may also become pregnant. Pregnancy is associated with profound cardiovascular changes in women, and pregnancy-related pathologies, such as hypertensive disorders of pregnancy, intrauterine growth restriction, and preterm birth, are associated with excess lifelong CVD risk (37). Given that BP tends to go back to the normotensive range, even after hypertensive disorders of pregnancy, and that preterm birth or intrauterine growth restriction can occur even when the mother is normotensive throughout pregnancy, BP alone might not identify women who are progressing toward CVD after an adverse pregnancy outcome (37). More sensitive measures of vascular function are warranted during and after pregnancy (38).

Best practices for collecting vascular structure and function measurements during reproduction have not been established. The current recommendations for assessment of vascular function in non-pregnant adults require an overnight fast preceding testing and specify that vascular testing should be performed with the participant in the supine position (36, 39). A lengthy fast might be an unreasonable request for a pregnant or breastfeeding woman, as pregnancy and breastfeeding are associated with heightened energy needs for the mother or baby. Further, maintaining blood sugar is important for promoting fetal growth, and morning sickness can be exacerbated by low blood sugar.

Pregnant women are advised to avoid lying supine to prevent the weight of the baby and uterus from compressing the vena cava. Lying supine for a 30–45 min testing session past the first trimester would not be acceptable for fetal health and might cause a drop in maternal BP. We propose the use of a reclining, padded table set at a standard 45° angle and conducting testing 1–2 h after a light meal that is low in vasoactive saturated fat and refined carbohydrates (40). The reproducibility of vascular function measurements in pregnant and breastfeeding women using these proposed methods has not yet been rigorously evaluated.

## Subfertility in Reproductive Aged Women and Vascular Structure and Function

Early menopause and conditions related to subfertility have also been associated with higher risk of overt CVD in women (41, 42). Anti-Müllerian hormone (AMH), a biomarker of ovarian reserve, is an established indicator of menopausal onset, and longitudinal data demonstrated that lower baseline and rapidly declining levels of AMH were individually associated with higher risks of CVD and coronary heart disease in women (41). Primary ovarian insufficiency (POI) is a form of early menopause that develops before the age of 40, is characterized by low AMH levels, high follicle stimulating hormone levels, amenorrhea, and hypoestrogenism, and is causally related to higher CVD risk in affected women (42). Women diagnosed with POI achieved fewer pregnancies and exhibited unfavorable lipid profiles and hypertension, and yet they exhibited similar coronary artery calcium scores compared to unaffected controls (42, 43). However, an inverse relationship between AMH levels and femoral and carotid intima-media thickness has been described in women with POI (20). There is little information regarding lifestyle behavior and subclinical atherosclerosis in women with POI.

Women diagnosed with polycystic ovarian syndrome (PCOS), an endocrine disorder characterized by high AMH levels, hyperinsulinemia, amenorrhea or oligomenorrhea, and hyperandrogenism, also present with unfavorable lipid profiles, hypertension, subfertility, higher fasting insulin levels and arterial stiffness, and have a higher risk for developing CVD (21, 44). Studies have shown that carotid intima-media thickness is higher in women with PCOS compared to age and BMI-matched controls, thus it may be a useful measurement for defining subclinical atherosclerotic progression and may serve as an outcome for lifestyle interventions in this population (22). A clinical trial investigated the effect of metformin on vascular function in women with PCOS and found that arterial stiffness, augmentation index, and endothelial function were all improved after a 12-week course of metformin (23). As lifestyle modification has been shown to be more effective than metformin for preventing cardiometabolic disease in middle aged adults (26), future lifestyle interventions in younger women with PCOS should also test for changes in vascular function.

## Discussion and Summary of Research Needs

Given their generally healthy traditional CVD risk factor profile, sensitive assessments of vascular structure and function are needed to evaluate the success of lifestyle interventions in premenopausal women. Our understanding of CVD development and progression is lacking in women. Tracking changes in vascular function in response to lifestyle interventions, as well as evaluating associations of vascular structure and function with easily measured sex-specific biomarkers, will critically inform preventive and treatment strategies and risk stratification in women. Cycling hormone levels, OCP use, pregnancy and breastfeeding, and conditions linked to subfertility present challenges to achieving reproducible vascular measurements in premenopausal women and/or caveats for the application of findings of smaller studies. Rather than ignoring potentially important factors related to ovarian hormone levels or excluding women from studies, we suggest that investigators carefully consider these variables when planning research and systematically fill in the knowledge gap. Our specific recommendations for research are as follows:

- Sensitive measures of vascular structure and function should be included in clinical trials involving different types of lifestyle behaviors, i.e., resistance interval and endurance exercise training, weight loss, in premenopausal women.
- Studies should account for the menstrual cycle phase and perform vascular data collection during the early follicular phase when possible.
- Investigators should gather information regarding type and length of oral contraceptive use to use in main analyses or sensitivity analyses.
- Vascular measurement protocols for data collection during pregnancy and post-partum, including meal timing and body posture, must be developed and standardized.
- More lifestyle-based clinical trials should be conducted in women with POI and PCOS to better understand the role of lifestyle in mitigating the excess CVD risk observed with these conditions.

## Data Availability Statement

The original contributions presented in the study are included in the article/supplementary material, further inquiries can be directed to the corresponding author/s.

## Author Contributions

AL-C and SB drafted the manuscript, prepared the table, and critically edited the manuscript. All authors contributed to the article and approved the submitted version.

## Conflict of Interest

The authors declare that the research was conducted in the absence of any commercial or financial relationships that could be construed as a potential conflict of interest.
